# Stage-dependent responses to emergent habitat heterogeneity: consequences for a predatory insect population in a coffee agroecosystem

**DOI:** 10.1002/ece3.1161

**Published:** 2014-07-23

**Authors:** Heidi Liere, Ivette Perfecto, John Vandermeer

**Affiliations:** 1 Department of Biology, University of the South735 University Ave, Sewanee, Tennessee, 37383; 2 School of Natural Resources and the Environment, University of Michigan440 Church St., Ann Arbor, Michigan, 48109; 3 Department of Ecology and Evolutionary Biology, University of Michigan830 N. University, Ann Arbor, Michigan, 48109

**Keywords:** Agroecology, ant–hemipteran mutualism, *Azteca instabilis*, *Azya orbigera*, *Coccus viridis*, myrmecophilous predator, ontogenetic niche shift, population persistence, stage-structured populations

## Abstract

Interactions among members of biological communities can create spatial patterns that effectively generate habitat heterogeneity for other members in the community, and this heterogeneity might be crucial for their persistence. For example, stage-dependent vulnerability of a predatory lady beetle to aggression of the ant, *Azteca instabilis,* creates two habitat types that are utilized differently by the immature and adult life stages of the beetle. Due to a mutualistic association between *A. instabilis* and the hemipteran *Coccus viridis –* which is *A. orbigera* main prey in the area – only plants around ant nests have high *C. viridis* populations. Here, we report on a series of surveys at three different scales aimed at detecting how the presence and clustered distribution of ant nests affect the distribution of the different life stages of this predatory lady beetle in a coffee farm in Chiapas, Mexico. Both beetle adults and larvae were more abundant in areas with ant nests, but adults were restricted to the peripheries of highest ant activity and outside the reach of coffee bushes containing the highest densities of lady beetle larvae. The abundance of adult beetles located around trees with ants increased with the size of the ant nest clusters but the relationship is not significant for larvae. Thus, we suggest that *A. orbigera* undergoes an ontogenetic niche shift, not through shifting prey species, but through stage-specific vulnerability differences against a competitor that renders areas of abundant prey populations inaccessible for adults but not for larvae. Together with evidence presented elsewhere, this study shows how an important predator is not only dependent on the existence of two qualitatively distinct habitat types, but also on the spatial distribution of these habitats. We suggest that this dependency arises due to the different responses that the predator's life stages have to this emergent spatial pattern.

## Introduction

The vast majority of animal communities are not randomly distributed. Rather they tend to have uniform, clustered, or patchy distributions, and the question of how these patterns emerge, as well as the consequences of their existence, is an exciting topic in ecological research. Although there is abundant research that shows that habitat heterogeneity promotes persistence of otherwise unstable systems, for example consumer–resource interactions (Bailey et al. 1962; Hassell and May 1974; Hassell et al. 1991; Bonsall et al. 2002), we know little how stage-structured predator populations, whose life stages have variable responses to different habitat types, are affected by a heterogeneous environment. In many situations, stage-related variation in responses to habitat type should be incorporated into consumer–resource models due to its great potential to have community-level consequences (Miller and Rudolf 2011). Vertebrates with complex life cycles and holometabolous insects are examples of organisms whose traits are sufficiently different among life stages to call for stage-structured analyses to truly understand their dynamics and the effect of those dynamics on the community in which the organism is embedded. Here, we show how a detailed understanding of the different life stages of an important predatory beetle is essential to comprehend how the clustered distribution of an aggressive arboreal ant affects the distribution of this predator.

Lady beetles are voracious and effective predators in many natural and agricultural systems and their persistence and distribution is especially important for the natural control of important hemipteran pests (Dixon 2000). In order to understand their population dynamics, it might prove essential to investigate the details of the different life stages because even though they mostly consume the same prey type throughout their life, lady beetle life stages have very different dispersal capabilities as well as vulnerability to starvation and natural enemies. For example, the fact that lady beetle prey are often ephemeral and patchily distributed (Hodek and Honek 1996; Dixon 2000; Seagraves 2009) suggests a high starvation potential for larvae that have limited dispersal capabilities, but not so for strong flying adults who can disperse over large distances looking for sparsely distributed prey.

Due to the relatively more persistent prey populations as well as competition- and enemy-free space (Bristow 1991; Mahdi and Whittaker 1993; Sloggett and Majerus 2000a), ant-tended prey colonies could potentially be ideal habitats for lady beetles, especially for poor-dispersing larvae. However, due to the elevated risk of ant attacks, most hemipteran natural enemies are forced away from ant-tended areas except in periods of severe prey scarcity (Way 1963; Sloggett and Majerus 2000b). This does not hold, however, for the few ladybird beetles (for review, see Majerus et al. 2006) and a handful of other hemipteran predators (syrphid flies, lacewings, lepidoptera larvae) and parasitoids that have evolved morphological, chemical, or behavioral modifications that render them immune or relatively tolerant of ant attacks (Eisner et al. 1978; Majerus 1989; Hübner 2000; Völkl 2001). Even though there is some evidence that shows that the distribution of these myrmecophilous predators is closely related to that of the ants that tend their prey (Völkl 1992, 1995), the question of how ant distribution would affect the distribution and persistence of myrmecophilous natural enemies when only one life stage is tolerant to ant attacks remains unanswered. This question is not only relevant for myrmecophilous predators, but for any organism whose different life stages have different and sometimes even opposite responses to different habitat types.

The coccidophagous lady beetle *Azya orbigera* (Coleoptera: Coccinellidae) is a voracious predator whose adults are vulnerable to the aggressive tree-nesting ant *Azteca instabilis* (Hymenoptera: Formicidae) (Liere and Larsen 2010), but whose larvae have waxy filaments that render them immune to ant attacks (Liere and Perfecto 2008). Larvae are thus able to prey upon the abundant ant-tended populations of *Coccus viridis,* (Hemiptera: Coccidae). Additionally, when living on ant-patrolled plants, these beetle larvae are also relatively free of natural enemies (Liere and Perfecto 2008). Thus, on the one hand, the risk of larval mortality is significantly lower in ant-tended areas. On the other hand, the obvious fitness advantage for females to oviposit in ant-tended areas might be outbalanced by the high risk of mortality due to ant attacks. Because of these conflicting effects, the resulting consequences of this spatially clustered mutualism on the abundance and spatial distribution of this important predator are unclear.

The main objective in this study was to determine how the different life stages of *A. orbigera* beetles are affected by the habitat heterogeneity created by *A. instabilis* given that beetles have easy access to ant-tended areas only during their larval stage. Specifically, we sought to determine how the presence and spatially clustered *A. instabilis–C. viridis* mutualism affected the distribution and abundance of adult and larval *A. orbigera* in a coffee farm in Chiapas, Mexico. Because the scale at which the lady beetles might respond to the spatial distribution of the mutualism was unknown, we first performed a large-scale survey on a 45-ha permanent plot, where we studied the distribution of *A. orbigera* larvae and adults in relation to areas with and without ant nests and to the size of the ant nests clusters. Second, we examined the distribution of lady beetles around all the shade trees in a 50 × 50 m quadrat in relation to the distance to a nearby nest cluster. Lastly, to understand the effect of ant nests on the beetle distribution on adjacent coffee bushes, we sampled beetle adults and larvae within a 5 meter radius of individual ant nests.

## Materials and Methods

### Study site

The study took place in an organic shade-grown coffee farm in Chiapas, Mexico (see Vandermeer and Perfecto 2006; for details of the farm and the 45-ha permanent plot).

### Study system

**Figure 1:**
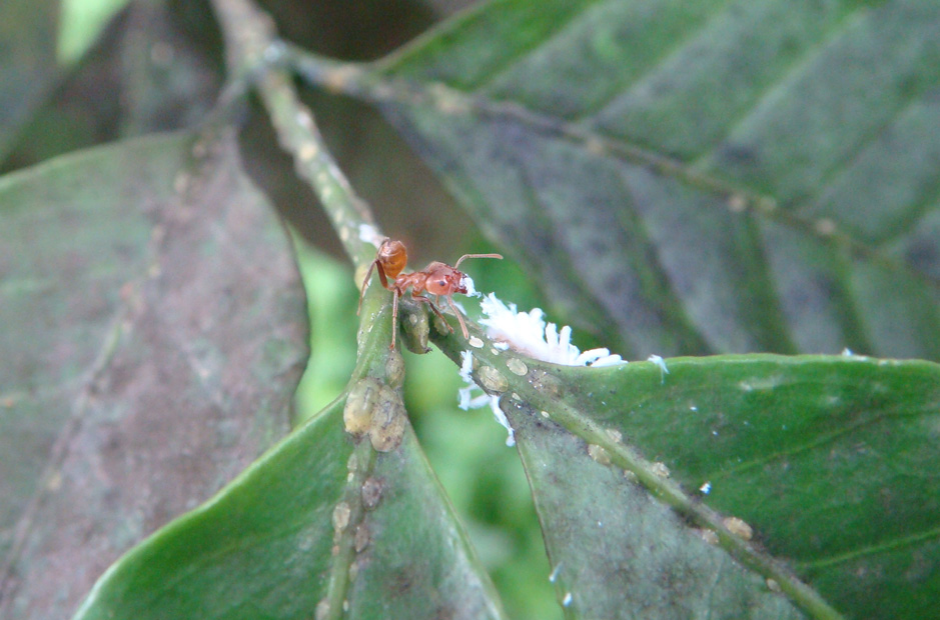
The image shows the three species in our study system: the *ant Azteca instabilis*, the hemipteran *Coccus viridis,* and the lady beetle larvae, *Azya orbigera* (behind the leaf). Waxy filaments of *A. orbigera* are stuck to the ant mandibles.

The ecological community under study consists of the hemipteran, *C. viridis*, its mutualistic tree-nesting ant, *A. instabilis*, and the predatory lady beetle, *A. orbigera* (Fig. 1). The mutualistic ant *A. instabilis* builds its nests in shade trees and tends *C. viridis* living on coffee plants in the surrounding two to three meter radius of the nest. Due to a combination of partial protection against natural enemies, improved hygienic conditions, and optimal feeding site selection mediated by *A. instabilis*, ant-tended *C. viridis* populations growth rate is higher than nontended populations (Jha et al. 2012) and, consequently, high densities (i.e., more than 50 scale per coffee bush) of *C. viridis* can almost exclusively be found in the protected radius around *A. instabilis* nests (Vandermeer and Perfecto 2006; Vandermeer et al. 2010). In an established 45-ha permanent plot in the coffee farm in Mexico, only approximately three percent of shade trees contain ant nests and these are distributed in a clustered form in space (Vandermeer and Perfecto 2006; Vandermeer et al. 2008). *Azya orbigera*, both as larvae and as adult, is a voracious predator of *C. viridis* and can eat an average of 20 individuals per day and, for larvae, this predation rate holds even when *C. viridis* is tended by ants (Liere and Perfecto 2008; Liere and Larsen 2010).

### Large scale: beetle distribution in a 45-ha plot in relation to ant nest presence and ant nest cluster size (45-ha plot)

To determine the large-scale lady beetle distribution in relation to ant nest presence and ant nest cluster size, we sampled coffee bushes around shade trees with and without ant nests. We superimposed a 50 × 50 m grid over the map of the 45-ha permanent plot. Given we knew the location of every *A. instabilis* nest in the plot (Vandermeer and Perfecto 2006; Vandermeer et al. 2008), we could determine whether each of the 50 × 50 m quadrants was occupied by ants or not. In the cases of quadrants without ant nests, we chose the centermost shade tree and searched the coffee bushes within a 5 m radius for *A. orbigera* adults and larvae. In the cases of quadrants with ant nests, we identified the centermost shade tree with an ant nest and sampled the coffee bushes using the same methodology described above. We excluded the quadrats on the edge of the plot from the survey. This survey was carried out twice in the rainy season (June/July 2006 and 2007) and twice in the dry season (January/February 2007 and 2008). During the first survey, there were a total of 55 quadrants with ants and 60 quadrants without ants, and 53 with and 63 without during the rest of the surveys. During the rainy season 2006, we recorded adult sex and larval instar. We excluded from the analysis trees that were cut or died during our study. For consistency with previous theoretical work on the system, we used *ant nest density* as a measure of ant nest clustering, estimated as the number of ant nests within a 20 m radius of each sampled point (Vandermeer and Perfecto 2006; Liere et al. 2012). However, given that sampled trees *without ants* had by our definition no ant nests within 20 m, to be able to explore ant nest clustering effects on beetle abundances in trees without ants, we also included the number of ant nests within 50 m as a covariate in the model.

### Data analysis

Given that a pre-analysis of the data from the first sampling season (rainy season 2006) showed no significant interactions between ant presence (and nest density) and adult sex or larval instar, we lumped all adult females and males into one category (adults) and all larval instars into another (larvae) for the analyses.

We used a generalized linear mixed-effect model (GLMM) to understand the effect of the explanatory variables on beetle abundance. We included *Site ID, season* (*dry/rainy*), and *sampling year* (years 1 and 2*)* as random terms in the model. We then used a backward selection process to find the optimal model by eliminating nonsignificant terms or interactions (Zuur et al. 2009) using the *lme4* package (Bates et al. 2011) for the R statistical programming language (R Development Core Team 2011). In the full model, we included (1) ant presence (with or without), (2) ant nest density at 20 m, and (3) ant nest density at 50 m, as fixed factors and the interaction between ant presence and ant nest density at 50 m. Despite the potential correlation between variables b and c, we chose to leave them in the initial full model because we believed they could have very different effects on beetle abundance. Furthermore, the *Variance Inflation Factors* (VIFs) for these variables were reasonable low (2.43 and 2.3, respectively), which indicates that there is not a strong collinearity among them and thus can be safely used for further analysis (Zuur et al. 2007).

### Medium scale: beetle distribution in relation to the distance from an ant nest cluster in a 50 × 50 m quadrant

Given that for the large-scale sample we only counted beetles around one tree in the middle of each of the 116 quadrants in the permanent plot, we did not capture the effect of ant nests on beetle distribution around adjacent shade trees to the ant nests. Thus, we selected a 50 × 50 m quadrant with a group of closely located ant nests and that were relatively isolated from other ant nests and sampled adult and larva beetles in the coffee bushes around all shade trees: for a total of seven trees with ant nests and 70 trees without ant nests. We performed one single sampling during the rainy season 2008, when we searched for beetles for 30 min on all coffee bushes within a five meter radius of each tree in the quadrant. The objective of this sample was to investigate beetle abundance as a function of the distance to the ant nest cluster.

### Data analysis

First, we ran a Kruskal–Wallis chi-squared test to compare beetle abundances in trees with and without ants. Then, to determine the beetle abundance in the quadrant (only in trees without ant nests) as a function of the mean distance to the ant nest cluster, we ran a generalized linear model with a Poisson distribution.

### Small scale: beetle distribution in coffee bushes within 5 m of ant nests

The next step in our study was to determine how beetle abundance was distributed on the coffee bushes in the immediate vicinity of ant nests. Because *A. instabilis* mostly forage within a 2–3 m radius around their nests, to capture the local effect of ant activity on beetle abundance both inside active foraging areas and its peripheries, we sampled beetles within 5 m from individual ant nests.

We randomly chose two groups of closely located ant nests (i.e., ant nest clusters) within our permanent plot. Group # 1 had a total of seven closely located ant nests (less than 20 m apart), and Group # 2 had nine nests. In the rainy season 2005, we conducted three 2-week-interval samplings (between July and August) around each nest within the two chosen groups. We counted the number of adults and larvae on each of the five most adjacent coffee bushes to the ant nest (most of which were 0–4 m away from the nest) for 5 min and spent an additional ten minutes looking for beetles in area between 4 and 5 m away from the tree (for a total of 35 min per nest). We annotated the distance from the nest at which each individual was found. Because of the proximity of the three sampling dates, for each nest, we averaged the number of individuals per distance range (0–1, 1–2, 2–3, 3–4, and 4–5 m away from the ant nest) for the three sampling dates.

### Data analysis

We ran a GLMM with beetle abundance as a function of distance range (0–1, 1–2, 2–3, 3–4, and 4–5 m) and included *cluster* as random term.

## Results

### Large scale: beetle distribution in a 45-ha plot in relation to ant nest presence and ant nest cluster size (45-ha plot)

**Figure 2:**
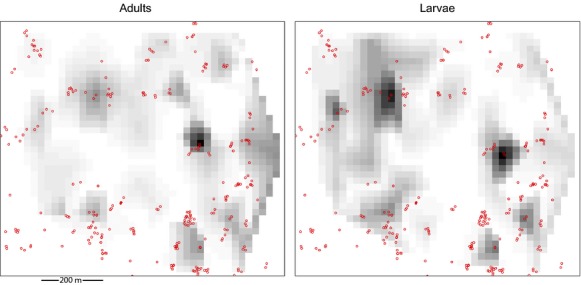
Map of a 45-ha permanent plot in a coffee farm in Mexico showing *Azya orbigera* abundance distribution in relation to *Azteca instabilis* nests. Red dots represent *A. instabilis* nests, and gray-scaled squares represent beetle adult (left panel) and larvae (right panel) abundance in coffee bushes within a 5 m radius of the sampled tree. White squares represent zeroes, light gray squares represent low abundances, and dark gray squares represent high abundances (adults: min = 0, max = 43; larvae min = 0, max = 56). One tree per 50 × 50 m quadrant was sampled. The maps show the sampling of rainy season, 2006.

Figure 2 shows the spatial distribution of ant nests and adult and larval beetle abundance during the rainy season of the first sampling year. We found that a GLMM with a random intercept and slope terms for site, year, and season, significantly reduced the AIC values (Table 1). Mean adult abundance increased from 0.4 individuals per focal tree (ind/tree) in areas without ants to 3.3 ind/tree in areas with ants, while mean larvae abundance increased from 0.4 ind/tree in areas without ants to 1.4 ind/tree in areas with ants (GLMM results: Tables 1, 2, 3). Ant nest density at 20 m had a significant positive effect on adult abundances but a negative albeit nonsignificant one on larvae abundance (Table 2). Ant nest density at 50 m was not present in the best models for adults or for larvae. The pseudo-*R*^2^ (estimated with a Spearman correlation of the fitted vs. observed values) was 0.51 for adults and for larvae 0.38.

**Table 1:** Generalized linear mixed-effect models fit comparisons for a 45-ha plot of *Azya orbigera* abundance as a function of the presence of *Azteca instabilis* nests and ant nest cluster size in a coffee farm in Mexico (see Materials and Methods for details on the models)

	Model	df	AIC	BIC	Residual deviance
Adults	1	8	1022.42	1054.9	1006
	2	14	968.85	1025.7	940.8
	3	13	966.87	1019.6	940.9
	4	12	964.87	1013.6	940.9
Larvae	1	8	839.3	871.7	823.3
	2	14	782.3	839.1	754.3
	3	13	780.6	833.3	754.6
	4	12	778.7	827.4	754.7

There were a total of 428 observations nested in 107 sites, 2 seasons, and 2 years.

Model 1: Beetle density − ant nest presence + ant nest density at 20 m + ant nest density at 50 m + ant presence × ant nest density at 50 m as fixed effects; *Site ID*, *year,* and *season* were random factors with random intercepts.

Model 2: same as model 1, but the random terms with random intercepts and random slopes.

Model 3: same as model 2, but eliminated the least significant fixed term (ant nest density at 50 m × ant presence interaction).

Model 4: same as model 3, but eliminating least significant interaction (ant nest density at 50 m).

**Table 2:** Results of the best generalized linear mixed-effect models (see Materials and Methods for details on the models) for different sampling scales of *Azya orbigera* beetles in relation to *Azteca instabilis* ant nests in a coffee farm in Mexico

		Adults			Larvae		
			
	Fixed effects	Coefficient	SE	*P*-value	Coefficient	SE	*P*-value
45-ha plot^1^	Intercept	−2.89	1.50	0.05	−7.21	3.86	0.06
	Ant nest presence (no–yes)	2.40	1.05	0.02	6.35	3.01	0.03
	Ant nest cluster size	0.11	0.06	0.06	−0.10	0.07	0.14
50 × 50 m plot	Intercept	2.44	0.4	<0.01	2.73	0.42	<0.01
	Mean distance to ant nest cluster	−0.11	0.02	<0.01	−0.13	0.02	<0.01
5 m sample	Intercept	−0.25	0.08	<0.01	0.25	0.06	<0.01
	Mean distance to ant nest cluster	0.22	0.03	<0.01	−0.05	0.02	0.02

1 For the 45-ha plot sample, there were a total of 428 observations nested in 107 sites, 2 seasons, and 2 years.

**Table 3:** Random factor coefficients for the generalized linear mixed-effect models (see methods for details on the models) for the 45-ha plot sample of *Azya orbigera* beetles in relation to *Azteca instabilis* ant nests in a coffee farm in Mexico

		Intercept		Ants	
			
	Random effects	Variance	SD	Variance	SD
Adults	Site	1.05	1.02	3.92	1.98
	Season	3.48	1.86	1.37	1.17
	Year	0.95	0.97	0.56	0.75
Larvae	Site	2.46	1.57	8.63	2.93
	Season	7.68	2.77	4.81	2.19
	Year	21.75	4.66	12.69	2.56

There were a total of 428 observations nested in 107 sites, 2 seasons, and 2 years.

### Medium scale: beetle distribution in relation to the distance from an ant nest cluster in a 50 × 50 m quadrant

**Figure 3:**
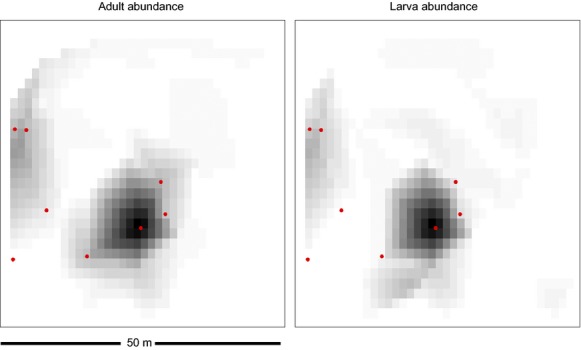
Map of 50 × 50 m quadrant in a coffee farm in Mexico showing *Azya orbigera* abundance distribution in relation to *Azteca instabilis* nests during the rainy season (June 2008). Red dots represent *A. instabilis* nests and black/gray-scaled squares represent beetle adult (left panel) and larvae (right panel) abundance in coffee bushes within a 5 m radius of the sampled tree (all shade trees in the quadrant were sampled). White squares represent zeroes, light gray squares represent low abundances, and dark gray squares represent high abundances.

Figure 3 shows the beetle distribution map in the quadrant in relation to the ant nest cluster. There were significant differences in the mean rank of beetles among sites with and without ants (adults: *H* = 18.61, df = 1; *P* = 1.6 × 10^−05^; larvae: *H* = 11.44, df = 1, *P* = 0.0007). The mean adult abundance per site was 19.1 (±6.68 SE, *n* = 8) in sites with ants and 1 (±0.28 SE, *n* = 70) in sites without ants. The mean larva abundance per site was 12.00 (±6.6 SE, *n* = 8) in sites with ants and 0.94 (±0.29 SE, *n* = 70) in sites without ants. There was a significant negative relationship between beetle abundance in trees without ants and the mean distance to all ant nests in the quadrant (adults pseudo-*R*^2^ = 0.10; larvae pseudo-*R*^2^ = 0.09; Table 2).

### Small scale: beetle distribution in coffee bushes within 5 m of ant nests

**Figure 4:**
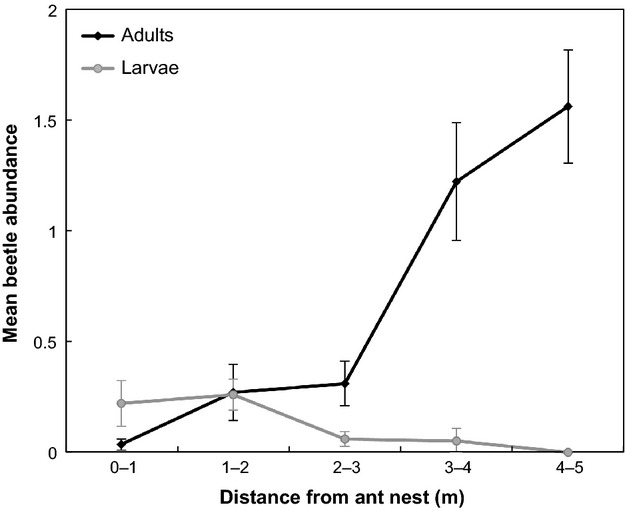
Relationship between *Azya orbigera* abundance within a 5 m radius of *Azteca instabilis* nests and the distance from the nest. The *y*-axis represents the mean abundance of beetles across 16 sampled ant nests and three sampling dates in the rainy season, 2005. For easier visualization, here we show the univariate relationship; the results for the generalized linear mixed-effect model can be found in the text.

Adults beetle abundance was higher with increasing distance from the nest (pseudo-*R*^2^ = 0.45), while larvae abundance decreased with increasing distance from the nest (pseudo-*R*^2^ = 0.09) (Table 2 for GLMM results; Fig. 4 shows the univariate response).

## Discussion

Explicitly considering demographic heterogeneity within populations might sometimes provide us with a better understanding of community structure and dynamic than unstructured approaches (Miller and Rudolf 2011). For example, ontogenetic niche shifts (i.e., changes in resource use, competitive ability, or vulnerability to consumption) can increase population persistence by reducing competition between stages (De Roos et al. 2003; De Roos and Persson 2013; Wollrab et al. 2013), by creating stage refugia that are invulnerable to consumers (Murdoch et al. 2003), or by avoiding resource overexploitation (Loreau and Ebenhoh 1994; Abrams and Quince 2005). Although it has been shown in theoretical studies that demographic heterogeneity plays an important role in community stability and persistence, there is still a shortage of empirical evidence showing how important this heterogeneity is in natural communities (Miller and Rudolf 2011). Our study provides empirical evidence of how ontogenetic differences in vulnerability in the face of an aggressive competitor can influence the population dynamics of an important predatory holometabolous insect.

The spatial distribution of myrmecophilous natural enemies, or natural enemies that have adaptations to overcome ant attacks, has been found to be closely associated with that of the ants that tend their prey (Völkl 1992, 1995). In our case study, however, because of the conflicting effects of the aggressive ant, *A. instabilis*, toward the different life stages of the lady beetle *A. orbigera*, it was unclear how this predatory lady beetle would respond to the spatially clustered distribution of the ant and its mutualistic partner, *C. viridis*, which is one of *A. orbigera* most important prey in the area (Vandermeer et al. 2010). We did find that, like other myrmecophilous natural enemies, the abundance and distribution of *A. orbigera* are closely related to that of the ants that tend their prey. Even though only larvae have protection against ant attacks, both larvae and adult beetles were significantly more abundant in areas tended by ants. However, after closer examination of the distribution of beetles in the immediate surroundings of ant nests, we found that, as expected, adults are restricted to the peripheries of areas of strong ant activity, while larvae are more abundant in the immediate vicinity of ant nests. Moreover, the effect of the clustered distribution of ant nests had a contrasting effect on the two life stages.

Contrary to most holometabolous insects that have distinct ecological life styles and exploit different resources as larvae and as adults (Truman and Riddiford 1999), lady beetles consume the same prey throughout their lives and are thus prone to strong intraspecific competition (Hodek and Honek 1996). Nevertheless, even though both beetle adults and larvae were more abundant in areas with ant nests, adults were restricted to the peripheries of highest ant activity and outside the reach of coffee bushes containing the highest densities of lady beetle larvae. Thus, *A. orbigera* does undergo an ontogenetic niche shift, not through shifting prey species, but through stage-specific vulnerability differences against a competitor that renders areas of abundant prey populations inaccessible for adults but not for larvae.

The greater abundance of lady beetle larvae in ant-tended coffee plants can be explained by the positive effect of *A. instabilis* on *A. orbigera* larvae survival (Liere and Perfecto 2008) and by the higher prey availability in these areas (Vandermeer and Perfecto 2006; Jha et al. 2012). In fact, inside ant-tended patches, lady beetle larvae thrive and exert a great predation pressure on ant-tended *C. viridis* (Liere and Perfecto 2008), sometimes to the point that *C. viridis* no longer appears to benefit from ant protection (Jha et al. 2012) and its populations are eventually diminished so much that the tending ant nest dies (Liere et al. 2012).

In contrast, adult lady beetles are restricted to the peripheries of ant-tending areas where prey availability is lower. Nevertheless, even though adult lady beetles are able to survive on alternative food (nectar, pollen, or alternative prey) in times of food scarcity (Hodek and Honek 1996), they need to feed on suitable or “essential” prey in order to reproduce (Hodek 1960; Triltsch 1999). Because adult lady beetles are very efficient in detecting individual prey (Hattingh and Samways 1992), they likely find the sparsely distributed *C. viridis* in the matrix of coffee bushes not protected by ants. Thus, the ontogenetic shift in vulnerability against ants creates a spatial heterogeneity for the lady beetle that may not only be responsible for their population persistence by reducing intraspecific competition, but also forces adults to disperse to rest of the farm, arguably contributing to maintaining *C. viridis* populations at low levels in the area (Liere et al. 2012). Similar ontogenetic niche partitioning may be true for other lady beetles, and using stage-structured approaches to study their population dynamics may lead to a better understanding of their persistence in agricultural systems and their effectiveness as biocontrol agents.

Interestingly, despite their inability to access ant-tended *C. viridis* colonies, *A. orbigera* adults tend to aggregate in areas around ant nests, probably waiting for opportunities to oviposit or feed on abundant ant-tended prey. Female beetles hide their eggs to protect them against ant predation by ovipositing on old *A. orbigera* pupal cases that still have the waxy filaments or under dead *C. virids* (Hsieh et al. 2012), a lengthy process that may be facilitated by a natural enemy of *A. instabilis*, specifically a parasitoid fly. In a series of studies at this site, the presence of the phorid fly, *Pseudacteon* spp. (Diptera: Phoridae) drastically reduced ant activity for at least two hours (Philpott et al. 2004; Philpott 2005) and allowed adult beetles to prey upon ant-tended *C. viridis* in experimental settings (Liere and Larsen 2010). Phorid presence may thus allow adult beetles to feed on abundant prey colonies, but more importantly, to oviposit in areas where their larvae will have abundant food and reduced mortality due to natural enemies. Because *A. orbigera* females are attracted by alarm pheromones released by *A. instabilis* to indicate phorid presence (Hsieh et al. 2012), it is possible that due to greater phorid activity in larger ant nest clusters (Vandermeer et al. 2008), female beetles might favor larger clusters to oviposit. Accordingly, adult beetles, but not larvae, were more abundant in larger clusters. The same phorid-induced reduction in ant activity that favors adult beetles in larger ant nest clusters might have a slight negative effect on larvae by temporarily leaving them vulnerable to natural enemies and thus neutralizing the higher oviposition rates in larger clusters.

The nature of our 45-ha sampling, that is, one sampled tree in the middle of each of the 50 × 50 m quadrants, made it impossible for us to evaluate the effect of ant nest clustering, measured as the number of nests within a 20 m radius, on beetles in areas without ants. Consequently, we added the number of nests within a 50 m radius of the sampled tree to our analyses. However, the latter clustering variable was not significant in any of the models, suggesting that either (1) beetles are only able to perceive ant nest clustering at a very local scale, or (2) they prefer only groups of very closely located nests, (3) beetles in areas without ants do not respond to ant nest cluster size. Nevertheless, in the 50 × 50 m sampling, the effect of an ant nest cluster on beetle distribution on surrounding trees was significant. Here, both larvae and adult beetles were more abundant and closer to the nest cluster. As explained above, adults are attracted to ant nest clusters and it is likely that if they do not manage to oviposit inside the ant activity area, they would prefer to do so as close as possible to the ant nests, thus the greater larvae abundance in nontended coffee plants closer to the ant nest cluster.

After a superficial examination, this system could be seen as a community composed of an aggressive and superior competitor (*A. instabilis*) and a nonaggressive and inferior competitor (*A. orbigera*) exploiting the same resource (*C. viridis*), a system which could easily lead to the extinction of the inferior competitor. However, a closer examination including the demographic heterogeneity allowed us to see that the aggressive competitor is only superior to one of the life stages of its nonaggressive competitor. The differential competitive abilities of the inferior competitor's life stages effectively create a spatial separation that may very well contribute to its population persistence. These effects are further complicated by the fact that the “superior” competitor is not a competitor for and actually benefits the larval life stage of the “inferior” competitor (Liere and Perfecto 2008; Liere and Larsen 2010). Simulation studies of the same system (Liere et al. 2012) show that these interactions lead to interesting and unexpected dynamics. As suggested by recent theoretical studies (De Roos and Persson 2013; Wollrab et al. 2013), we believe that similar demographic details may explain the stability of other predator–prey or competitive systems with apparent inherent unstable interactions.

Thus, our results are not only relevant for myrmecophilous predators, but for any organism whose life stages have different and sometimes even opposite responses to different habitat types. We show how an ontogenetic niche shift in competitive ability can create a heterogeneous spatial distribution even when the predator does not undergo an ontogenetic prey shift. The two emergent habitat types (one is occupied mainly by larvae, and the other by adults) are both necessary for the predator populations and, furthermore, areas where both habitat types occur in close proximity (in our case, ant nest clusters) sustain higher predator populations than do areas where one habitat type is relatively rare (in our case, isolated ant nests). Thus, our results together with evidence presented elsewhere (Jha et al. 2012; Liere et al. 2012) show how an important predator is not only dependent on the existence of two qualitatively distinct habitat types but also on the spatial distribution of these habitats. We propose that in our system, this dependency arises due to the contrasting ways in which the predator's life stages interact with the mutualism between their prey and ants, and the consequent subtle stage-dependent spatial distribution differences with respect to the emergent habitat heterogeneity.

## References

[b1] Abrams PA, Quince C (2005). The impact of mortality on predator population size and stability in systems with stage-structured prey. Theor. Popul. Biol.

[b2] Bailey VA, Nicholson AJ, Williams EJ (1962). Interaction between hosts and parasites when some host individuals are more difficult to find than others. J. Theor. Biol.

[b3] Bates D, Maechler M, Bolker B (2011). http://CRAN.R-project.org/package=lme4.

[b4] Bonsall MB, French DR, Hassell MP (2002). Metapopulation structures affect persistence of predator-prey interactions. J. Anim. Ecol.

[b5] Bristow CM (1991). Are ant-aphid associations a tritrophic interaction? Oleander aphids and Argentine ants. Oecologia.

[b6] De Roos AM, Persson L (2013). Population and community ecology of ontogenetic development.

[b7] De Roos AM, Persson L, McCauley E (2003). The influence of size dependent life history traits on the structure and dynamics of populations and communities. Ecol. Lett.

[b8] Dixon AFG (2000). Insect predator-prey dynamics: ladybird beetles & biological control.

[b9] Eisner T, Hicks K, Eisner M (1978). “Wolf-in-Sheep's-Clothing” strategy of a predaceous insect larva. Science.

[b10] Hassell M, May R (1974). Aggregation of predators and insect parasites and its effect on stability. J. Anim. Ecol.

[b11] Hassell M, May R, Pacala S (1991). The persistence of host-parasitoid associations in patchy environments. I. A general criterion. Am. Nat.

[b12] Hattingh V, Samways MJ (1992). Prey choice and substitution in *Chilocorus* spp. (Coleoptera: Coccinellidae). Bull. Entomol. Res.

[b13] Hodek I (1960). The influence of various aphid species as food for two lady-birds *Coccinella 7-punctata* L. and *Adalia bipunctata* L.

[b14] Hodek I, Honek A (1996). Ecology of Coccinellidae.

[b15] Hsieh HY, Liere H, Soto EJ, Perfecto I (2012). Cascading trait-mediated interactions induced by ant pheromones. Ecol. Evol.

[b16] Hübner G (2000). Differential interactions between an aphid endohyperparasitoid and three honeydew-collecting ant species: a field study of *Alloxysta brevis*. J. Insect. Behav.

[b17] Jha S, Allen D, Liere H, Perfecto I, Vandermeer J (2012). Mutualisms and population regulation: mechanism matters. PLoS ONE.

[b18] Liere H, Larsen A (2010). Cascading trait-mediation: disruption of a trait-mediated mutualism by parasite-induced behavioral modification. Oikos.

[b19] Liere H, Perfecto I (2008). Cheating on a mutualism: indirect benefits of ant attendance to a coccidophagous coccinellid. Environ. Entomol.

[b20] Liere H, Jackson D, Vandermeer J (2012). Ecological complexity in a coffee agroecosystem: spatial heterogeneity, population persistence and biological control. PLoS ONE.

[b21] Loreau M, Ebenhoh W (1994). Competitive exclusion and coexistence of species with complex life cycles. Theor. Popul. Biol.

[b22] Mahdi T, Whittaker B (1993). Do Birch Trees (*Betula pendula*) grow better if foraged by wood ants?. J. Anim. Ecol.

[b23] Majerus MEN (1989). *Coccinella magnifica* (Redtenbacher): a myrmecophilous ladybird. Br. J. Entomol. Nat. Hist.

[b24] Majerus MEN, Sloggett JJ, Godeau JF, Hemptinne JL (2006). Interactions between ants and aphidophagous and coccidophagous ladybirds. Popul. Ecol.

[b25] Miller TEX, Rudolf VHW (2011). Thinking inside the box: community-level consequences of stage-structured populations. Trends Ecol. Evol.

[b26] Murdoch WW, Briggs CJ, Nisbet RM (2003). Consumer-resource dynamics.

[b27] Philpott SM (2005). Trait-mediated effects of parasitic phorid flies (Diptera: Phoridae) on ant (Hymenoptera: Formicidae) competition and resource access in coffee agro-ecosystems. Environ. Entomol.

[b28] Philpott SM, Maldonado J, Vandermeer J, Perfecto I (2004). Taking trophic cascades up a level: behaviorally-modified effects of phorid flies on ants and ant prey in coffee agroecosystems. Oikos.

[b29] R Development Core Team (2011). R: a language and environment for statistical computing.

[b30] Seagraves MP (2009). Lady beetle oviposition behavior in response to the trophic environment. Biol. Control.

[b31] Sloggett JJ, Majerus MEN (2000a). Aphid-mediated coexistence of ladybirds (Coleoptera: Coccinellidae) and the wood ant *Formica rufa*: seasonal effects, interspecific variability and the evolution of a coccinellid myrmecophile. Oikos.

[b32] Sloggett JJ, Majerus MEN (2000b). Habitat preferences and diet in the predatory Coccinellidae (Coleoptera): an evolutionary perspective. Biol. J. Linn. Soc.

[b33] Triltsch H (1999). Food remains in the guts of *Coccinella septempuctata* (Coleoptera: Coccinellidae) adults and larvae. Eur. J. Entomol.

[b34] Truman JW, Riddiford LM (1999). The origins of insect metamorphosis. Nature.

[b35] Vandermeer J, Perfecto I (2006). A keystone mutualism drives pattern in a power function. Science.

[b36] Vandermeer J, Perfecto I, Philpott SM (2008). Clusters of ant colonies and robust criticality in a tropical agroecosystem. Nature.

[b37] Vandermeer J, Perfecto I, Philpott SM (2010). Ecological complexity and pest control in organic coffee production: uncovering an autonomous ecosystem service. Bioscience.

[b38] Völkl W (1992). Aphids or their parasitoids: who actually benefits from ant-attendance?. J. Anim. Ecol.

[b39] Völkl W (1995). Behavioral and morphological adaptations of the coccinellid, *Platynaspis luteorubra* for exploiting ant-attended resources (Coleoptera: Coccinellidae). J. Insect Behav.

[b40] Völkl W (2001). Parasitoid learning during interactions with ants: how to deal with an aggressive antagonist. Behav. Ecol. Sociobiol.

[b41] Way MJ (1963). Mutualism between ants and honeydew-producing Homoptera. Ann. Rev. Entomol.

[b42] Wollrab S, De Roos AM, Diehl S (2013). Ontogenetic diet shifts promote predator-mediated coexistence. Ecology.

[b43] Zuur AF, Ieno EN, Smith GM (2007). Analysing ecological data.

[b44] Zuur AF, Ieno EN, Walker NJ, Saveliev AA, Smith GM (2009). Mixed effects models and extensions in ecology with R.

